# Design and 3D Printing of Personalized Hybrid and Gradient Structures for Critical Size Bone Defects

**DOI:** 10.1021/acsabm.3c00107

**Published:** 2023-04-18

**Authors:** Mine Altunbek, Seyedeh Ferdows Afghah, Ali Fallah, Anil Ahmet Acar, Bahattin Koc

**Affiliations:** † Nanotechnology Research and Application Center, Sabanci University, Istanbul 34956, Turkey; ‡ Faculty of Engineering and Natural Sciences, Sabanci University, Istanbul 34956, Turkey; § Integrated Manufacturing Technologies Research and Application Center, Sabanci University, Istanbul 34906, Turkey; ∥ University of Massachusetts Lowell, Lowell, Massachusetts 01854, United States

**Keywords:** 3D printing, hybrid printing, gradient structures, large bone defects, personalized scaffolds

## Abstract

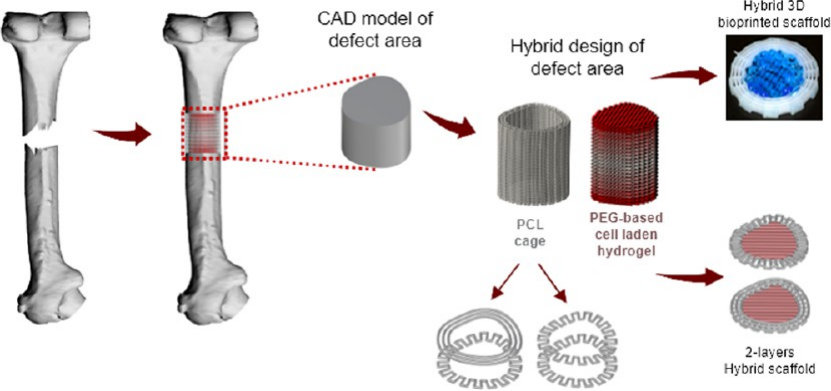

Treating critical-size bone defects with autografts, allografts, or standardized implants is challenging since the healing of the defect area necessitates patient-specific grafts with mechanically and physiologically relevant structures. Three-dimensional (3D) printing using computer-aided design (CAD) is a promising approach for bone tissue engineering applications by producing constructs with customized designs and biomechanical compositions. In this study, we propose 3D printing of personalized and implantable hybrid active scaffolds with a unique architecture and biomaterial composition for critical-size bone defects. The proposed 3D hybrid construct was designed to have a gradient cell-laden poly(ethylene glycol) (PEG) hydrogel, which was surrounded by a porous polycaprolactone (PCL) cage structure to recapitulate the anatomical structure of the defective area. The optimized PCL cage design not only provides improved mechanical properties but also allows the diffusion of nutrients and medium through the scaffold. Three different designs including zigzag, zigzag/spiral, and zigzag/spiral with shifting the zigzag layers were evaluated to find an optimal architecture from a mechanical point of view and permeability that can provide the necessary mechanical strength and oxygen/nutrient diffusion, respectively. Mechanical properties were investigated experimentally and analytically using finite element analysis (FEA), and computational fluid dynamics (CFD) simulation was used to determine the permeability of the structures. A hybrid scaffold was fabricated via 3D printing of the PCL cage structure and a PEG-based bioink comprising a varying number of human bone marrow mesenchymal stem cells (hBMSCs). The gradient bioink was deposited inside the PCL cage through a microcapillary extrusion to generate a mineralized gradient structure. The zigzag/spiral design for the PCL cage was found to be mechanically strong with sufficient and optimum nutrient/gas axial and radial diffusion while the PEG-based hydrogel provided a biocompatible environment for hBMSC viability, differentiation, and mineralization. This study promises the production of personalized constructs for critical-size bone defects by printing different biomaterials and gradient cells with a hybrid design depending on the need for a donor site for implantation.

## 1 Introduction

Bone plays a critical role in serving a number of functions in the human body including supporting the body’s skeleton and facilitating movement, protecting internal organs, and mineral storage.^1,2^ Their intrinsic self-regeneration capacity with well-regulated intracellular and extracellular signaling between the osteoprogenitor cells controls bone tissue regeneration during injury or remodeling throughout the lifespan.^3^ The self-renewal capability, on the other hand, becomes limited in critical-size injuries, which necessitates a surgical operation to support the defect area for regeneration.^4^ Allo- and auto-bone grafts are gold standards for implantation, but these have limitations and drawbacks.^5−8^ Allografts could be prepared with desired shapes but the lack of vascularization hinders their integration with the host tissue.^9^ Moreover, they might trigger an immunogenic response or cause disease transmission. Autografts are vascularized structures and include mesenchymal stem cells, osteoblasts, and osteoclasts, which can integrate better with the host tissue and fasten bone regeneration.^10−12^ However, autografts might not provide enough structural support, which results in miss-anastomosis and nonhealing deformities in clinical applications.^8,12^ In addition, both allo- and autografts could significantly decrease the patient’s life quality and cause complications after the surgical operation which might necessitate a secondary surgery, additional bone grafting, or amputation of the limb.^8^ Thus, there is an urgent need for alternative repair or substitution grafts for critical-size bone defects.

The advancement in tissue engineering with three-dimensional (3D) printing technology promises the biofabrication of mechanically supportive and biologically active structures for large bone defects specific to patients. Polycaprolactone (PCL),^13−15^ polylactic acid (PLA),^16−18^ polyglycolic acid (PGA),^19^ or poly lactic acid-*co*-glycolic acid (PLGA)^20−22^ are commonly used as synthetic biocompatible and biodegradable thermoplastic polymers for the construction of mechanically strong scaffolds for critical-size bone defects. Fused deposition modeling (FDM) or extrusion-based 3D printing and melt electrospinning writing (MEW) approaches are commonly used for these types of polymers.^14,23−25^ With the coordinated deposition of the microfilaments, various scaffolds have been constructed with different sizes of interconnected pores and desired mechanical strength.^13,14^ These scaffolds can provide a 3D environment for cell migration, proliferation, and differentiation as well as nutrient and oxygen transport and waste removal. Despite their advantages, these synthetic polymers cannot be employed as bioinks for cell bioprinting and hence limit their applications for tissue engineering. Thus, further improvements in terms of the structure and biochemical composition are required for a better and accelerated healing process.

Fabrication of biomimetic functional tissuelike structures has excellent potential for efficient bone regeneration.^5^ By integrating various bioprinting technologies, bioactive scaffolds can be fabricated via a combination of mechanically strong thermoplastic scaffolds with homogeneous cell-laden matrixes.^26^ Hydrogels are capable of meeting the requirements of bioprinting considering their hydrophilic nature with a loose and permeable structure that provides a cell-friendly environment enabling better cell migration and differentiation.^27^ A number of natural and synthetic hydrogels such as gelatin, collagen, alginate, and poly(ethylene glycol) (PEG) are used to encapsulate cells and bioactive molecules for the construction of functional and active scaffolds for bone tissue engineering.^26,28,29^ For example, in a study performed by Atala et al., a multicartridge 3D printing system was used to fabricate hybrid structures comprising cell-laden bioinks including gelatin, fibrinogen, hyaluronic acid, and glycerol with different cell types, and mechanically strong 3D printed PCL structures layer by layer for bone tissue engineering.^15^ Recent research by Hernandez et al.^30^ demonstrated a hybrid structure of a cell-laden hydrogel mixture consisting of alginate, gelatin, and nanohydroxyapatite injected into PCL for large bone defects. Three different geometries were investigated for the PCL structure including mesh with 0/90° layer deposition, honeycomb, and gyroid. The gyroid architecture had found with a higher hydrogel loading capacity with the ability to facilitate more vascularization, and mechanical properties were similar to trabecular bone tissue. Another hybrid structure was recently reported by Dubey et al., through the reinforcement of a bioactive magnesium phosphate-laden gelatin methacryloyl (GelMA) hydrogel within MEW fibrous PCL mesh structures for periodontal bone tissue regeneration.^31^ Their findings revealed that the proposed hybrid structure was promising for the whole regeneration process with optimized mechanical properties with the geometrical design of PCL mesh, and stimulated osteogenesis due to the presence of magnesium phosphate in GelMA. There are several examples of hybrid functional structures for bone tissue engineering,^32−37^ yet, the supporting layers are basically constructed by printing meshlike structures where cell-laden hydrogels are deposited in alternating layers. These structures are simple but lack the hierarchical structure of the defect area. In addition, the limited diffusion of oxygen and nutrients through the hydrophobic and solid matrix impairs the long-term function of living cells inside the hybrid structure. Another challenge with the existing methods is not being able to control cell and active biomolecule composition throughout the hybrid structures. Microcapillary extrusion printing has shown promising results in the highly controlled deposition of multicellular/biomaterials during printing for the fabrication of a gradient structure.^38,39^

In this study, we aimed to engineer and construct patient-specific implants in a novel biomimetic hybrid design and composition by using 3D printing technologies of melt extrusion-based and microextrusion capillary printing. The computer-aided algorithms were developed and printing paths and parameters were planned for the sequential deposition of porous PCL cages and a cell-laden hydrogel structure. The printing algorithm of PCL cages at different geometries was optimized experimentally and numerically using nonlinear finite element analysis (FEA) according to their mechanical properties. Furthermore, mass transport characterization such as permeability and wall shear stress (WSS) of the printed cages with different patterns was also investigated by a computational fluid dynamic (CFD) simulation in axial and radial directions to determine the most convenient design that facilitates the diffusion of gas and nutrients through the stiff structure. At last, the final scaffold with the optimized cage structure was fabricated through a hybrid printing approach of deposition of a PCL cage and bioprinting of human bone marrow mesenchymal stem cell (hBMSC)-laden PEG-based bioink in a layer-by-layer manner. A customized cell-laden bioink at various cell numbers was bioprinted to recapitulate the complexity and heterogeneity of the defect area of the donor, and the printability of the hybrid and gradient structures was demonstrated. The biocompatibility and osteoinductive capacity of the PEG-based hydrogel were evaluated by monitoring cell viability, ALP enzyme activity, and mineralization for their potential to differentiate into a bonelike structure. The results of this study reveal the strong potential of the hybrid and gradient design of the 3D bioprinted scaffolds for alternative personalized implantable scaffolds for critical-size bone defects.

## 2 Materials and Methods

### 2.1 Design and 3D Printing Topology for Personalized, Hybrid, and Gradient Bone Tissue Constructs

**Figure 1:**
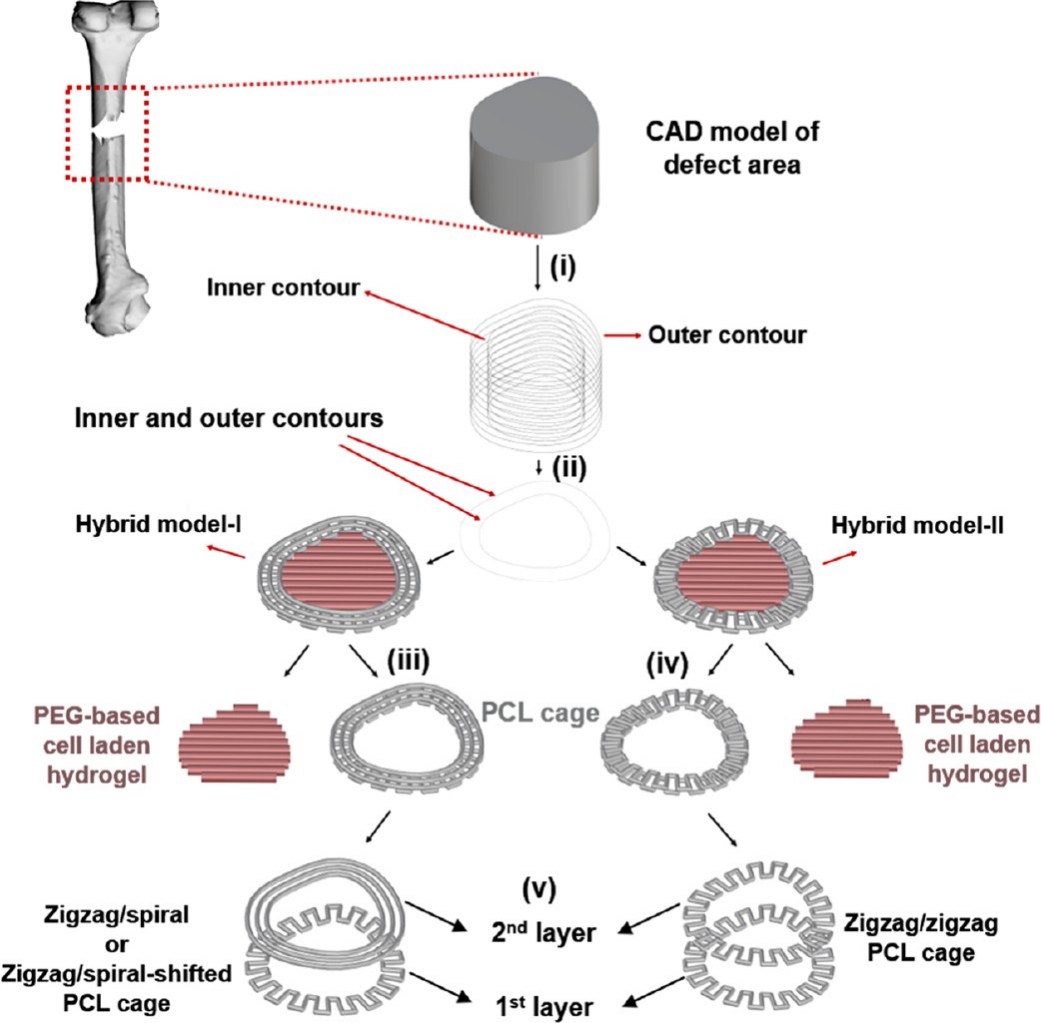
Schematic representation of CAD modeling and calculation of the 3D hybrid printing path using different PCL cage geometries. (i) Extracting a CAD model of the defect area, (ii) determining the inner and outer contours of the PCL cage, (iii) designing different models for the hybrid structure, (iv) sequential printing PCL and cell-laden hydrogel structures in the hybrid design, and (v) showing the first two layers of the PCL cage.

Three concentric porous structures including zigzag/zigzag, zigzag/spiral, and zigzag/spiral shifted were designed for the PCL cage architecture and evaluated with their mechanical strength and oxygen/medium diffusion capacity.^14,40,41^ A novel algorithm was developed to control the microarchitecture of the constructs and also continuously print directly from the medical images. Figure 1 represents the steps from modeling the defect area to path planning for PCL cages and hybrid structure designs. First, a magnetic resonance (MR) image of the patient who suffered from large bone defects was used for modeling patient-specific structures. The 3D images were first converted into stereolithography (STL) files to have information on the object’s surface geometry. The process is followed by sectioning of these STL files into 2D layers from which the models are created.^42^ The inner and outer contours were calculated to reach the same porosities for these different models. The algorithm creation was briefly as follows: (i) Inner and outer contours of the PCL cage were created using the obtained CAD model (parametric surface). (ii) Three alternative designs were determined for the PCL cage structure: (iii) Zigzag/spiral: outer and inner contours were divided into equal intervals (interval length parameter) and by combining these points alternatively, the first layer was created, offsetting these two contours, and calculating the spiral printing path in the second layer. One zigzag pattern and one spiral pattern were repeated in the alternating layers. Zigzag/spiral-shifted: For this design, one zigzag pattern and one spiral pattern were used, but the second zigzag layer was started in the opposite direction of the first zigzag layer. (iv) Zigzag/zigzag: outer and inner contours were evenly divided. The first layer was created by connecting the obtained points with each other alternatively. In the second layer, the points were connected to each other in the opposite direction of the first layer. The same printing pattern was repeated every two layers. This model was denoted as the zigzag model. In each layer, PCL and the cell-laden hydrogel were printed separately by sequentially utilizing melt extrusion printing and microcapillary-based bioprinting. (v) The first two layers of the PCL cage architecture is demonstrated.

The bioprinting path for the cell-laden PEG-based bioink was also planned as mesh structures in the center of the PCL cage with no gap distance between deposited filaments. The printing path planning codes were run to extrude the PEG-based bioink continuously inside the PCL cage. The printing path was continued until the inner circle of the PCL cage was completely filled.

### 2.2 Extrusion 3D Printing of PCL Cages

PCL cages (CAPA 6400 Perstorp, U.K.) with zigzag, zigzag/spiral, and zigzag/spiral-shifted geometries were fabricated using a custom-made extrusion-based 3D printing platform. In this 3D printer system, the print head can move in three axes while the collector platform is fixed. For 3D printing of PCL cages, a 10 mL metal syringe connected to a 250 μm diameter precision nozzle (Musashi Inc., Japan) is mounted on the print head with a heating system (New Era Pump Systems, Inc.). The feeding system works with a pneumatic regulator (Musashi Inc., Japan) that connects to the syringe. PCL pellets were added into the syringe and the metal syringe was heated to 80 °C and waited for an hour. The applying pressure was adjusted to 2.9–3.5 bar with printing speeds of 75–100 mm/min to deposit the melt PCL fibers with different geometries. 34, 20, and 21 points were determined for the inner and outer contours to construct different patterns with similar porosities. The inner and outer diameters were 8 and 5 mm, respectively.

### 2.3 Structural Characterization of the 3D Printed Scaffolds

Microcomputed tomography (μ-CT) is an effective tool for the 3D structural characterization of complex and porous geometries.^43^ We visualized the whole 3D structure of the 3D printed PCL cages via a high-resolution μ-CT (SkyScan 1172, Bruker, Belgium) and calculated their porosities. The 3D printed structures were scanned with a source voltage and current of 62 kV, and 159 μA, respectively. The images were taken with angular steps of 0.7°, and rotation steps of 0.19°, 5 average frames with no filter. NRecon software (version 1.5) was used to reconstruct the data with 20% of beam hardening correction and a ring artifact correction of 6. The images were then analyzed by CT-Analyzer v1.19.4.0 software (CTAn, Bruker, SkyScan, Belgium), and the total porosities of the scaffolds were identified. The actual porosities of the 3D printed structures were also calculated experimentally according to the following equation
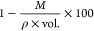
1where *M* is the mass of the printed structure (kg), ρ is the density of PCL (kg/m^3^), and vol. is the volume of the bounding box (m^3^).

### 2.4 Experimental and Numerical Analyses for Mechanical Properties of the 3D Printed PCL Structures

Mechanical properties of the 3D printed PCL cages with different architectures were examined experimentally and analytically. For the experimental analysis, a Zwick/Roell-Z100 universal testing machine (UTM) was used. The tests were carried out in the absence of the hydrogel to solely observe the effect of geometries. The structures were fabricated in hollow cylindrical shapes with the same level of total porosities. The sample size had 5 cm height, 4 cm wall thickness, and 7.5 cm and 12.5 cm inner and outer diameters, respectively. The compression tests were performed with a 100 kN static load cell at a velocity of 5 mm/min up to 35% of deformation. The force and deformation for each test were recorded, and the stress–strain curves were subsequently acquired based on the initial scaffold dimensions. The compression moduli and energy absorption were acquired by calculating the slope of the linear region, and the area under the curve of the stress–strain graphs. Each test was repeated at least four times and the results were presented as the average.

FE analysis determines the compression stiffness of the scaffolds. For FE analysis, the mechanical properties of the printed PCL fibers are needed. PCL shows an elastic–plastic behavior at room temperature.^44^ Here, the well-known Ludwik’s equation is used to describe the plastic behavior of the material
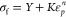
2where *K*, *Y*, and *n* are the strength coefficient, initial yield stress (Pa), and the strain-hardening exponent, respectively. The PCL material properties are extracted from our previous work.^41^ The CAD models are imported to Ansys Workbench (Ansys Inc., 2020) for the subsequent simulation. Two rigid plates are added to the top and bottom of the scaffolds. For the compression test simulation, one of the plates is considered fixed, and the other moves downward to exert the compression forces on the scaffolds. The contact between the rigid plates and scaffolds is fractional, with a friction coefficient of 0.3.

After the simulation, the reaction forces on the bottom plate are extracted. The theoretical effective compression moduli (*E*_theory_) can be calculated from the following relation
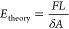
3where *F*, *L*, δ, and *A* are the reaction force (N), scaffold height (5.2 mm), applied displacement (0.52 mm), and cross-sectional area of the scaffold, respectively (73.9 mm^2^).

### 2.5 Permeability Analysis through Mass Transport Characterization of the 3D Printed PCL Structures

The fluid flow within the printed porous scaffolds is investigated by steady-state CFD analysis using Ansys workbench software. Based on the CFD results, it is possible to calculate the permeability and WSS, which play a vital role in characterizing the scaffolds’ biological behavior. Since the scaffolds have a different response in the radial and axial directions, the fluid flow in both directions is studied. In most of the previous studies in the literature, the permeability in the axial direction, i.e., the height of the scaffold, is studied.^45−47^ However, the permeability in the radial direction plays a critical role in the diffusion of the blood and nutrition to the tissue inside the PCL cage.

**Figure 2:**
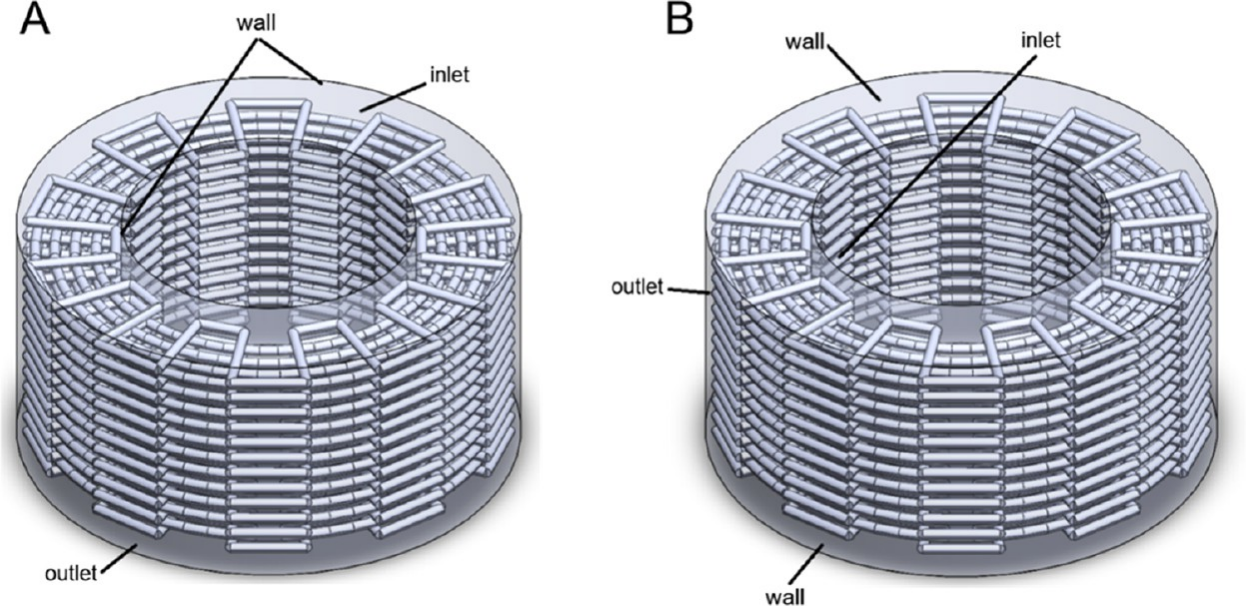
Fluid domain for the CFD simulation and boundary conditions for the (A) axial flow and (B) radial flow.

Figure 2 shows the fluid domain and the boundary conditions considered in the CFD simulation for both axial and directional flow. Additional void areas at the top, bottom, inner, and outer faces of the model decrease the boundary influence on the results. The fluid velocity at the inlet section is set to be 0.001 m/s regarding the bone *in vivo* conditions,^48,49^ while the gauge pressure at the outlet is set as zero. Assuming that the surfaces of scaffolds are hydrophilic, no-slip boundary conditions can be applied on the scaffold walls.^48^ In the simulation, a fluid with a density of 1000 kg/m^3^, and dynamic viscosity of 0.0037 Pa·s similar to cell culture media with 5% wt/wt Dextran is considered.^50^

Scaffold permeability, k, can be obtained by Darcy’s law as^51^
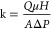
4in which *Q*, *H*, *A*, Δ*P*, and μ are the inlet fluid flow rate (m^3^/s), the distance between the inlet and outlet (m), the cross section area (m^2^) of the inlet, pressure drop (Pa), and dynamic viscosity coefficient (Pa·s) of the fluid, respectively. The fluid flow-induced WSS also can be computed as^52^
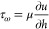
5where ω can be the *x*-, *y*-, and *z*-directions.

To reduce the computational cost, here 1/4 of the model is used. Whole models are meshed using tetrahedral elements.

### 2.6 Cell Culture

hBMSCs were purchased from ATCC (ATCC, PCS-500-012) and grown in the basal media (ATCC, PCS-500-030) supplemented with a growth kit for bone marrow-derived MSCs (PCS-500-041). Cells were maintained in a 37 °C, 5% CO_2_ supplemented humidified incubator and passaged when they reached 80–90% confluency. Passage numbers 4–5 (P4–5) were used for osteogenic differentiation experiments.

### 2.7 Bioink Preparation and Capillary-Based 3D Bioprinting of the Cell-Laden PEG-Based Bioink

Multiarm poly(ethylene glycol) (4-arm PEG vinylsulfone, PEG-VS, MW: 20 kDa, 95% VS modification, Jenkem Technology) was used to generate the bioink, as described previously.^39^ Briefly, 4-arm PEG was first functionalized with the RGD peptide (Ac-G**C**GYG**RGD**SPG-NH_2_) and then a peptide cross-linker specific to matrix metalloproteinase for bone formation (Ac-GCRE**GPQG↓IWGQ**ERCG-NH_2_) was used to cross-link the PEG via the Michael-type addition reaction. Since the reaction begins in a few seconds after the peptide cross-linker is added to the bioink mixture, each component for a defined volume of the bioink was aliquoted to have final concentrations as follows: PEG (6.5%, w/v), RGD peptide (200 μM), and peptide cross-linker (balanced with a stoichiometric amount of free −VS groups). The samples were freeze-dried and stored at −20 °C until use.

The bioprinting process of the PEG-based bioink was followed, as described in our previous study.^39^ A microcapillary based modified bioprinter system (Organovo) was used for the bioprinting of the PEG-based bioink. A printing head equipped with glass microcapillaries was used to aspirate the bioink. Just before the aspiration, the bioink was prepared as follows: For a 30 μL of the reaction volume of the bioink, the RGD peptide aliquot was dissolved in 3 μL of HEPES buffer (0.3 M, pH 8) at room temperature (RT) and added into PEG solution prepared in 7 μL of the same buffer. After adding the RGD peptide solution to the PEG solution, the final mixture was incubated for at least 30 min at 37 °C to functionalize the PEG macromers with the RGD peptide. Meanwhile, low-molecular-weight gelatin (LMWG) solution was dissolved in HEPES buffer at a concentration of 6% and placed at 37 °C until further use. Then, the PEG-RGD and LMWG solutions were equilibrated to RT. Meanwhile, hBMSCs were trypsinized and 5 μL of the cell suspension was mixed with PEG-RGD solution in final concentrations of 1 × 10^6^, 2 × 10^6^, and 5 × 10^6^ cells/mL in the final bioink solution. The peptide cross-linker aliquot was dissolved in 15 μL of 6% LMWG solution at RT. The resulting peptide cross-linker-LMWG mixture (with a final volume of 15 μL) was mixed with the PEG-RGD-cell mixture (with a final volume of 15 μL) at RT to obtain 30 μL of the final sample bioink mixture (PEG-RGD-cross-linker-LMWG mixture). Since the cross-linking reaction started as soon as this final mixture was prepared, the bioprinting process was started immediately by aspirating the bioink into 85 mm long and 500 μm in diameter microcapillaries at RT. After incubation of the microcapillary at 37 °C in the heat block reservoir for 4 min, the cylindrical PEG-based hydrogel stripes were dispensed in the computationally designed patterns. The bioprinted constructs were submerged in a growth medium and incubated at 37 °C in a humidified atmosphere containing 5% CO_2_.

### 2.8 Characterization of 3D Bioprinted Structures

#### 2.8.1 Cell Viability Analysis

Calcein AM/PI (live/dead) staining was performed on the bioprinted constructs on days 1, 3, and 7 to demonstrate the biocompatibility of the bioprinting process. Briefly, the samples were washed with 1× PBS at the end of the time points and transferred to glass-bottom Petri dishes. The constructs were incubated with 1 μM Calcein-AM (Invitrogen) in 1× PBS for 20 min at 37 °C. Next, the samples were washed three times with 1× PBS and incubated with 0.75 μM propidium iodide (PI) (Invitrogen) in 1× PBS for 5 min at 37 °C. After incubation, the samples were washed three times with 1× PBS, and the live/dead cells were visualized using an inverted confocal microscope (Zeiss LSM 710) at maximum excitation/emission wavelengths of 488/515 and 561/625 nm, respectively. The 3D image was obtained by scanning the 3D bioprinted constructs through a depth of ∼500 μm at 4.75 μm intervals on the *z*-axis.

#### 2.8.2 Alkaline Phosphatase (ALP) Activity

The bioink precursor solutions with 1 × 10^6^, 2 × 10^6^, and 5 × 10^6^ cells/mL cell densities were prepared, as described in Section 2.7. Monolayer hydrogel structures were bioprinted by smooth extrusion of polymerized PEG-based bioink fibers through the glass microcapillaries in a geometry that filled the PCL cage (Figure 8B) The cells were incubated in ATCC’s recommended medium (MM) for 3 days. Then, the osteocyte differentiation medium (OM) (ATCC) was replaced with the MM. The control groups were incubated in MM. Every 3–4 days, the medium was changed with fresh medium.

The differentiation of hBMSCs into bone tissue was evaluated by monitoring the alkaline phosphatase (ALP) activity. After 7, 14, and 21 days of incubation, 100 μL of M-PER Mammalian Protein Extraction Reagent (Thermo Fisher Scientific) was added to the 3D bioprinted constructs and incubated at room temperature. After 30 min of incubation, the constructs were centrifuged at 14 000 rpm for 15 min, and the supernatant was collected into a new tube. A BCA protein test kit was used to analyze the total amount of extracted protein. ALP activity was determined by measuring the 405 nm absorbance of the product formed after incubation with the 4-nitrophenyl phosphate (DNPP) substrate (Sigma-Aldrich) for 60 min at 37 °C. ALP activity was calculated by normalizing with the values per unit protein content obtained from the BCA test. At least three replicates were used for each time point.

#### 2.8.3 Alizarin Red Staining

The mineralization in the 3D bioprinted constructs was analyzed using alizarin red staining. The mono- and three-layered 3D structures were prepared by deposition of PEG bioinks through microcapillaries with final 1 × 10^6^, 2 × 10^6^, and 5 × 10^6^ hBMSCs densities in alternating layers. The structures were treated with OM for 21 days. After fixation with 4% paraformaldehyde for 30 min, the 3D bioprinted constructs were then stained with alizarin red solution (pH: 4.1–4.2) (Sigma) at a concentration of 2 mg/mL for 1 min. A serial washing step with distilled water was applied to remove the excess alizarin red dye from the constructs. The monolayer hydrogel structures were examined with a 10× objective lens under an inverted light microscope (Carl Zeiss Microscopy). The cross sections of three-layered gradient structures were examined under a stereomicroscope (Carl Zeiss Microscopy).

## 3 Results and Discussion

### 3.1 Design and 3D Printing Structures for Gradient Bone Tissue Constructs

The geometric design determines two critical properties of the PCL cage structure: (i) mechanical strength that must be strong enough to hold the load-bearing force applied to the defect area, and (ii) the porosity that has to supply the sufficient amount of the nutrients and oxygen to the cells encapsulated into the PEG-based hydrogel placed inside the PCL cage. Moreover, the pore geometries of this zigzag/spiral structure guided cells to fill the pores in a pattern that can help gradient mineralization in a shorter period of time.^41^ Hence, we decided to alternate three different models of zigzag structures: (1) zigzag, (2) zigzag/spiral, and (3) zigzag/spiral-shifted.

To generate the computational model of a personalized, hybrid, and gradient bone tissue construct, we started from an MR image of damaged critical-size bone tissue to calculate the 3D printing topology and determined the steps of the algorithm. To detect the best scaffold’s architecture, we investigated their mechanical properties both experimentally and analytically as well as their permeability through a mass transport simulation.

### 3.2 3D Printing of PCL Cages and Their Structural and Mechanical Analyses

3D printing of PCL cages was carried out using the melt extrusion printing technique. The patterns were designed to reach similar porosities for all three models. PCL pellets were poured into the metal syringe and heated to 80 °C and kept for 1 h at that temperature. Printing parameters were optimized and the porosity values of the printed structures were measured experimentally using eq 1 and compared to the values in the CAD models. The inner and outer diameters were 13 and 3 mm for all the PCL structures, respectively. The printing parameters are shown in Table 1.

**Table 1:** Printing Parameters of the 3D Printed PCL Cages

structure	applied pressure (bar)	printing speed (mm/min)	layer number
zigzag	2.8	80	22
zigzag/spiral	3.2	95	23
zigzag/spiral-shifted	3.5	110	24

**Figure 3:**
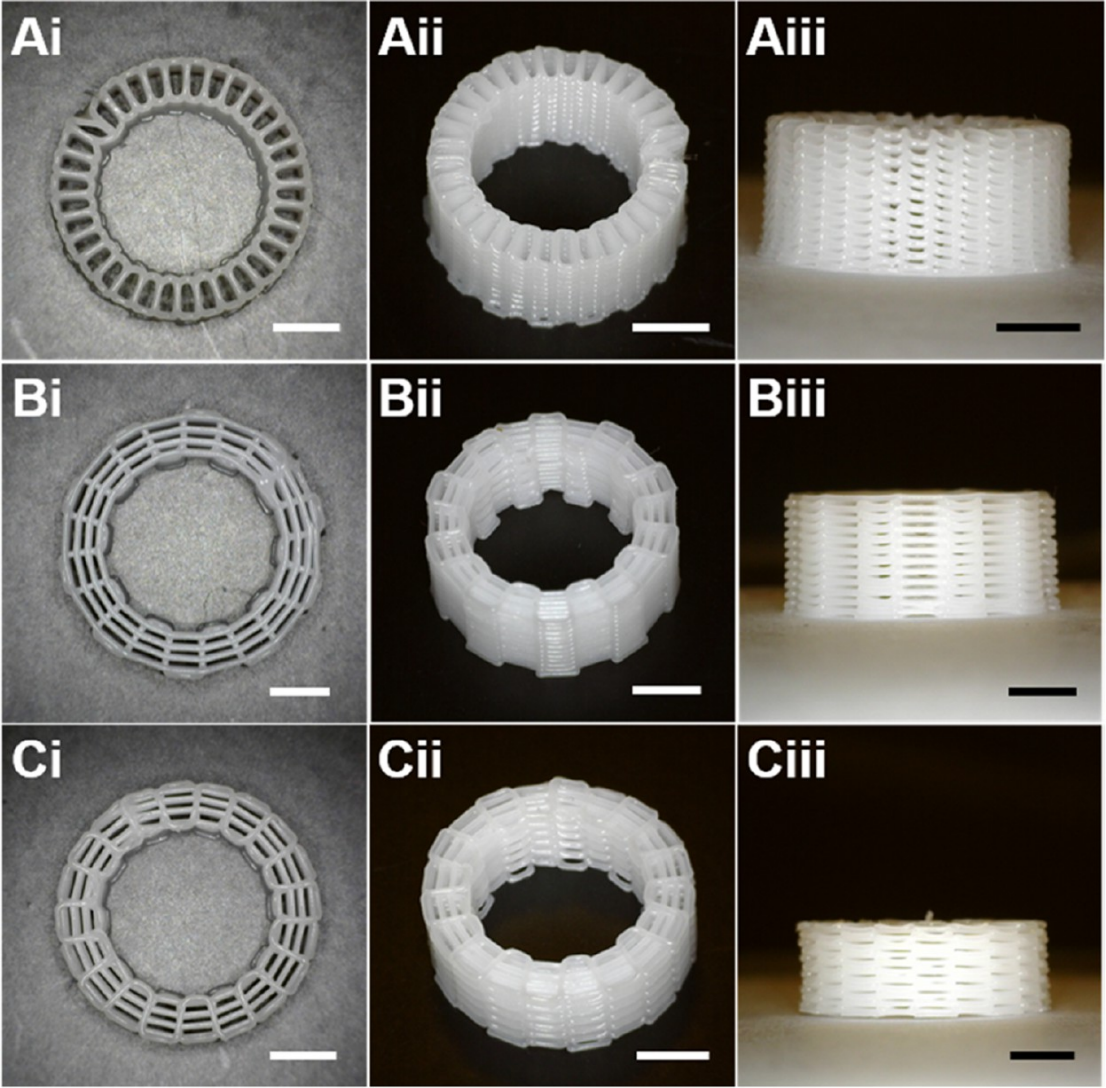
Digital images of the 3D printed PCL cages of (A) zigzag, (B) zigzag/spiral, and (C) zigzag/spiral-shifted patterns at (i) top view, (ii) perspective angle, and (iii) side view. Scale bars are 3 mm.

Following the printing, the structures were visualized at different angles using a high-resolution digital microscope (Dino-lite AM4113TR4, Taiwan), presented in Figure 3.

**Figure 4:**
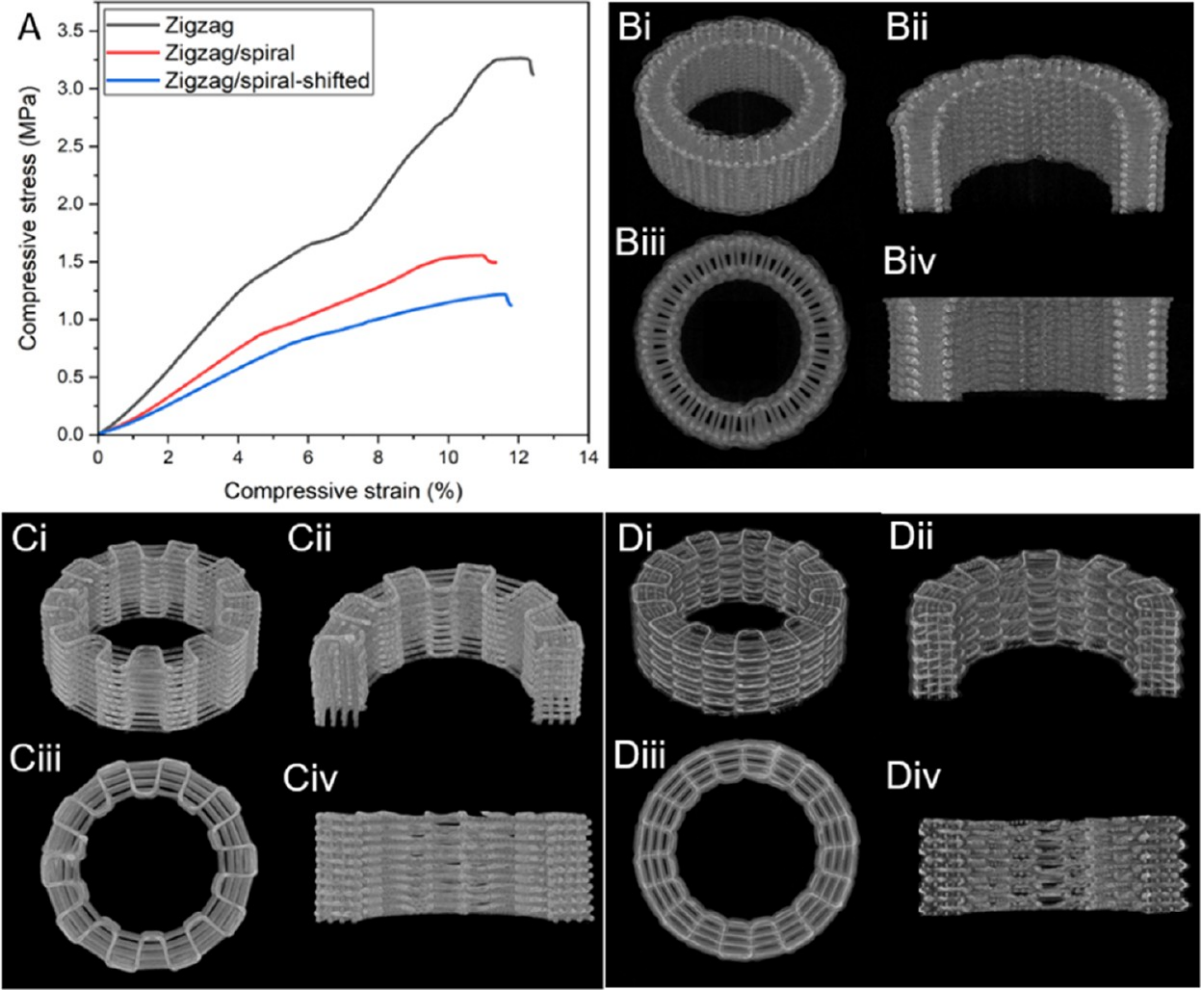
Characterization of the mechanical properties and porosity of the alternative PCL cage structures. (A) Compressive stress–strain curves for the 3D printed structures of zigzag, zigzag/spiral, and zigzag/spiral-shifted patterns. Micro-CT images of (B) zigzag, (C) zigzag/spiral, and (D) zigzag/spiral-shifted structures at a (i) perspective angle, (ii) cross-sectional perspective angle, (iii) top view, and (iv) cross-sectional side view.

The final design of the PCL cage was selected among alternative zigzag models based on the mechanical and permeability properties. First, we performed the compression test on the three different patterns of PCL cages to investigate the effect of their structural design on their mechanical properties. Figure 4 shows the stress–strain curves of the three structures and as the figure implies, all of the samples experienced a nonlinear behavior at the beginning of the compression test, followed by a linear pattern representing the elastic behavior. The compressive stiffness values were obtained by calculating the slope in this region. By increasing the compression force, the structures started to deform and showed plastic deformation. The zigzag structure showed the highest compressive strength of 32.88 ± 1.82 MPa compared to the other two zigzag/spiral patterns. For this pattern, the filaments were printed in a way that each layer was supporting the upper layer. The zigzag/spiral structures, on the other hand, have interconnection points between zigzag and spiral layers, which create weaker points under compression. The same pattern was observed for the yield point representing the starting stress that the structure showed plastic deformation. The yield point value was the lowest for the zigzag/spiral-shifted and the highest for the one with no spiral layer. In addition, it can be seen that the zigzag structure showed a higher stress amount revealing that it could bear more load under pressure at a specific deformation value.

Another indicator of how the scaffolds can withstand the applied force is the amount of energy absorbed during the compression test. In this context, energy absorption was also measured by calculating the area under the stress–strain curve. Table 2 shows the energy absorption of the structures, and the energy absorption capacity of the zigzag structure is clearly higher than those of the other two patterns.

**Table 2:** Mechanical Properties and Porosity Values of the 3D Printed Structures^a^

	compressive stiffness (MPa)			total porosity (%)	
structure	theoretical	experimental	energy absorption (kJ/m^3^)	theoretical	experimental
zigzag	33.59	32.88 ± 1.82	225.19 ± 6.37	67.24	66.12 ± 6.82
zigzag/spiral	20.51	20.65 ± 0.82	176.8 ± 10.71	70.94	69.04 ± 7.82
zigzag/spiral-shifted	15.71	15.39 ± 0.66	144.5 ± 8.07	68.10	66.26 ± 5.05

a Theoretical amounts of the compressive stiffness and total porosities were obtained from FEA simulation and Micro-CT analyses, respectively. Experimental porosities were calculated using eq 1.

The 3D scanned images from μ-CT are shown in Figure 4B–D revealing the interconnectivity of the pores with no flaws. The total porosity values of the PCL cage structures were calculated both theoretically by using μ-CT and experimentally using formula 1 (Table 2). The results showed that the total porosities obtained from the models were also close to the calculated values from μ-CT (data not shown) and all of the structures had almost the same amount of porosity, which eliminates the effect of porosity on mechanical properties.

FE analysis was used to calculate the effective compression stiffness and the stress distribution in the scaffolds with different patterns and to validate mechanical test results. The PCL constitutive model parameters extracted from the compression test are shown in Table 3 as the mean ± standard deviation (SD).

**Table 3:** PCL Properties for FEA Simulation

Young’s modulus (MPa)	yield strength (MPa)	yield strain (mm/mm)	strength coefficient (MPa)	strain hardening exponent
148.80 ± 12.73	10.22 ± 1.34	7.15 ± 1.33	34.28 ± 1.51	0.16 ± 0.02

**Figure 5:**
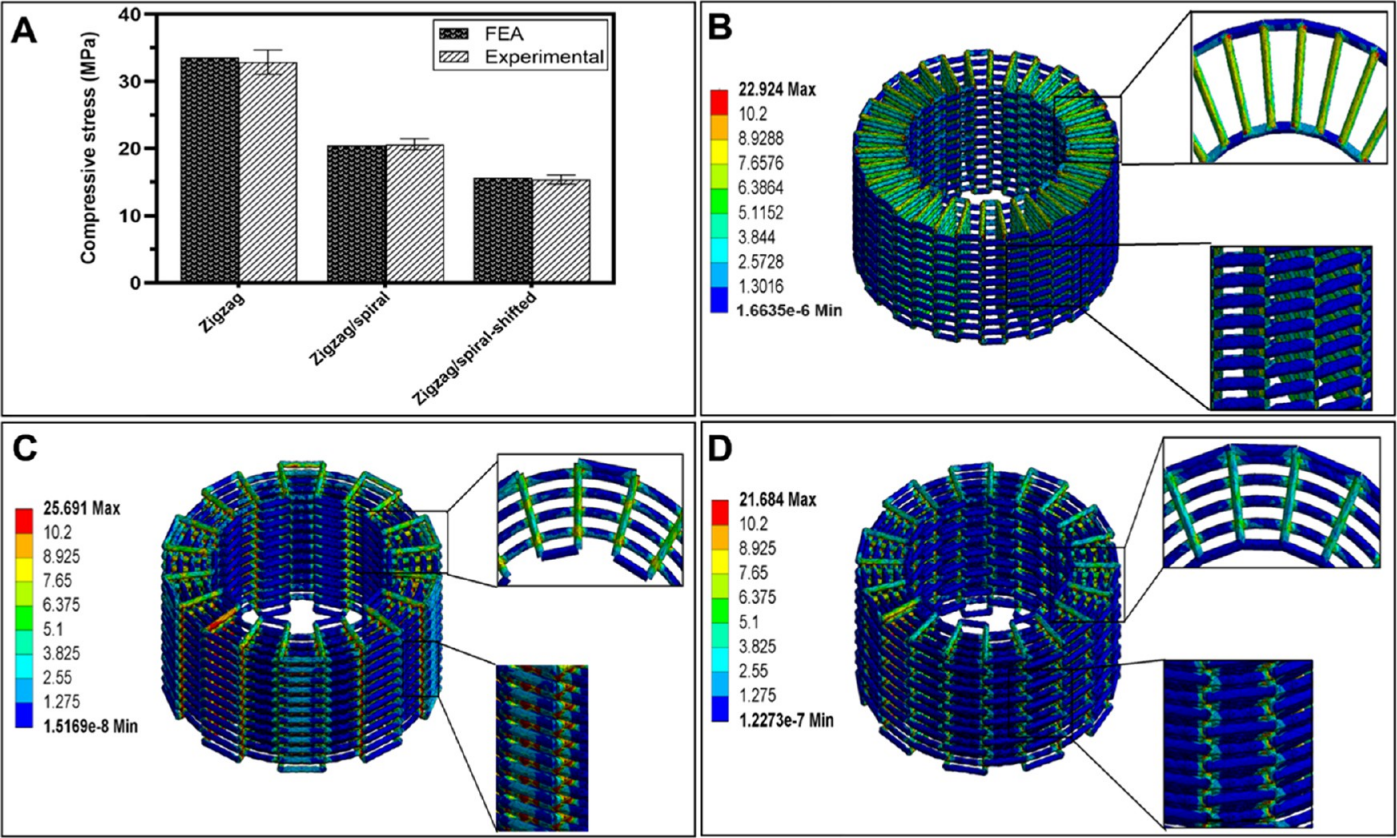
Mechanical strength analysis for the PCL scaffolds. (A) Comparison of the FE analysis and experimental compressive stress values of the 3D printed PCL scaffolds. von Mises stress distribution of the scaffolds with (B) zigzag, (C) zigzag/spiral, and (D) zigzag/spiral-shifted patterns.

Figure 5A shows the experimentally measured effective compressive stress and the simulation predictions for the different scaffolds. The zigzag pattern had a higher compressive stress than the zigzag/spiral and zigzag/spiral-shifted patterns although they had the same level of total porosity. It is known that in the 3D printed structures, the exerted loads are distributed and bear through filament junctions of the vicinity layers.^41^ In the zigzag pattern, the filaments of each layer supported the upper layers, and the contact area of the filaments in the alternating layers was higher compared to the other structures resulting in stiffer mechanical properties. Nonlinear FEA simulation can predict scaffold behavior better than the linear one. Table 2 represents the experimental and simulated compressive moduli of the PCL cages and as can be seen, the predicted values were consistent with the experimental ones.

The spatial distribution of stress in the scaffolds under loading for the three patterns is shown in Figure 5C,D. In this figure, different pseudo colors represent different stress levels. The structure’s lowest and highest stress levels are presented by blue and red colors, respectively. To better compare the patterns in all nephograms, stress levels higher than the yield point of PCL are represented by red color. The filaments at the junctions were almost in the plastic region for all patterns. The results showed that the stiffness of scaffolds with the zigzag pattern was around 50% higher than those with zigzag/spiral and zigzag/spiral-shifted patterns with the same porosity level. Moreover, the zigzag/spiral pattern had around 30% higher stiffness than the zigzag/spiral-shifted pattern. It should be noted that FE simulation results show that considering the elastic–plastic behavior of PCL leads to the prediction of the stiffness of the scaffolds with adequate accuracy.

### 3.3 Characterization of Mass Transport of the 3D Printed PCL Scaffolds

**Figure 6:**
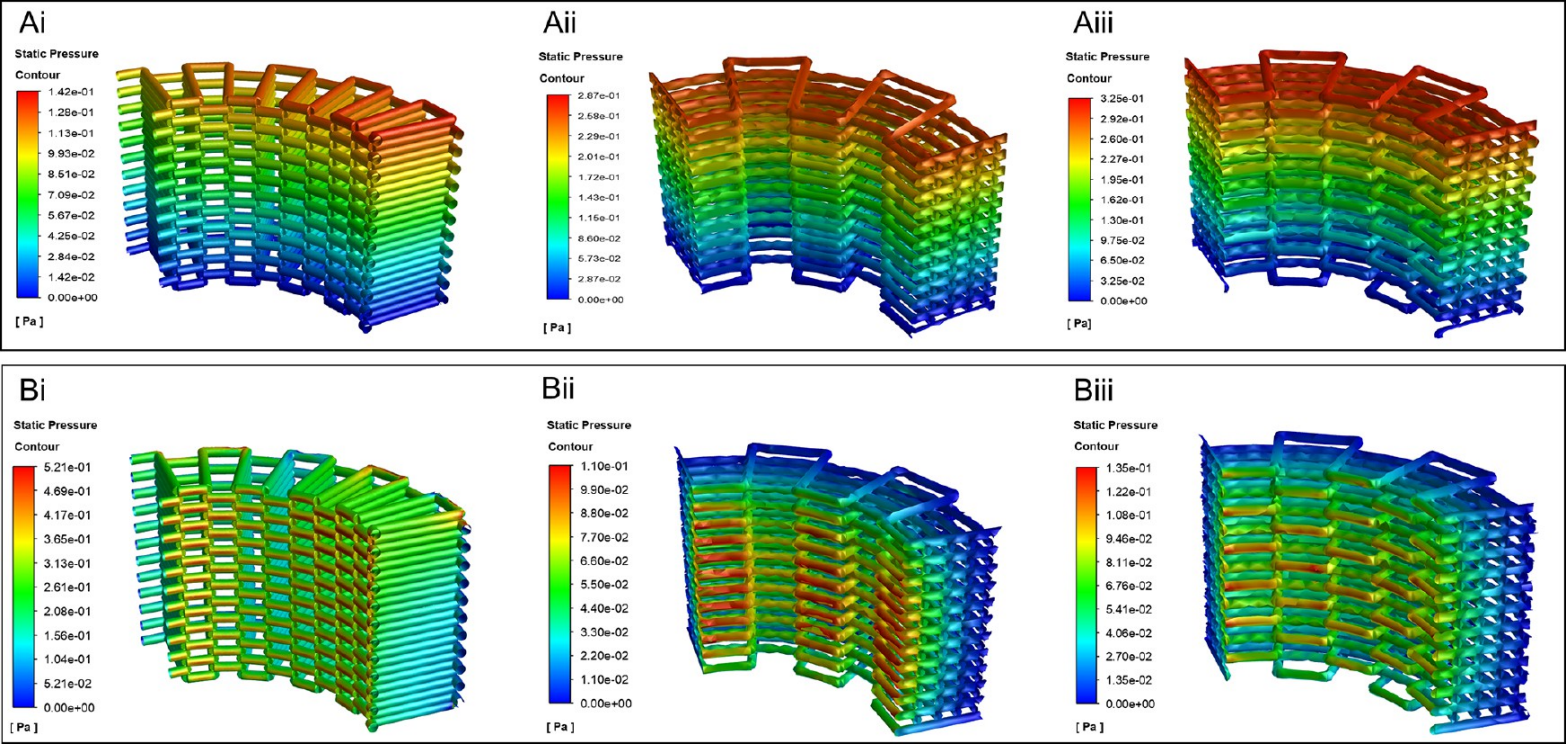
CFD simulation for the mass transport at (A) axial and (B) radial pressure distribution contours of the 3D printed scaffolds of (i) zigzag, (ii) zigzag/spiral, and (iii) zigzag/spiral-shifted patterns.

CFD simulation results for the mass transport characterization are presented in terms of permeability and the WSS inside the different scaffolds. The pressure distributions for all patterns were analyzed in both axial and radial flow directions and the results are presented in Figure 6. Because of the applied boundary condition, the highest pressure is at the inlet section, and it gradually tends to zero in the outlet section for all of the patterns. Based on the results, the zigzag/spiral-shifted pattern shows the highest pressure, and the zigzag shows the lowest one in the axial flow. However, in the radial flow, the zigzag pattern has the highest pressure, while the pressures in zigzag/spiral and zigzag/spiral-shifted are almost the same. These results can be interpreted considering the microstructure of the scaffolds in the radial and axial directions. For instance, in the zigzag pattern, the filaments are placed in a way that the flow’s path in the radial direction is blocked, while in the axial direction, there is no significant barrier in front of the flow. That is why the zigzag pattern has the highest permeability in the axial direction, but it has the lowest permeability in the radial direction. The results are presented in Table 4. Moreover, since the zigzag/spiral and zigzag/spiral-shifted microstructure in the radial direction is similar, their permeability in the radial direction is almost equal; however, in the axial direction, the zigzag/spiral has higher permeability than the zigzag/spiral-shifted.

**Table 4:** Wall Shear Stress and Permeability of the Scaffolds with Different Patterns

	axial		radial	
pattern	average WSS (mPa)	permeability (10^–9^ × m^2^)	average WSS (mPa)	permeability (10^–9^ × m^2^)
zigzag	3.11	15.66	14.7	3.08
zigzag/spiral	4.30	7.44	3.42	11.80
zigzag/spiral-shifted	5.42	6.22	3.07	11.76

**Figure 7:**
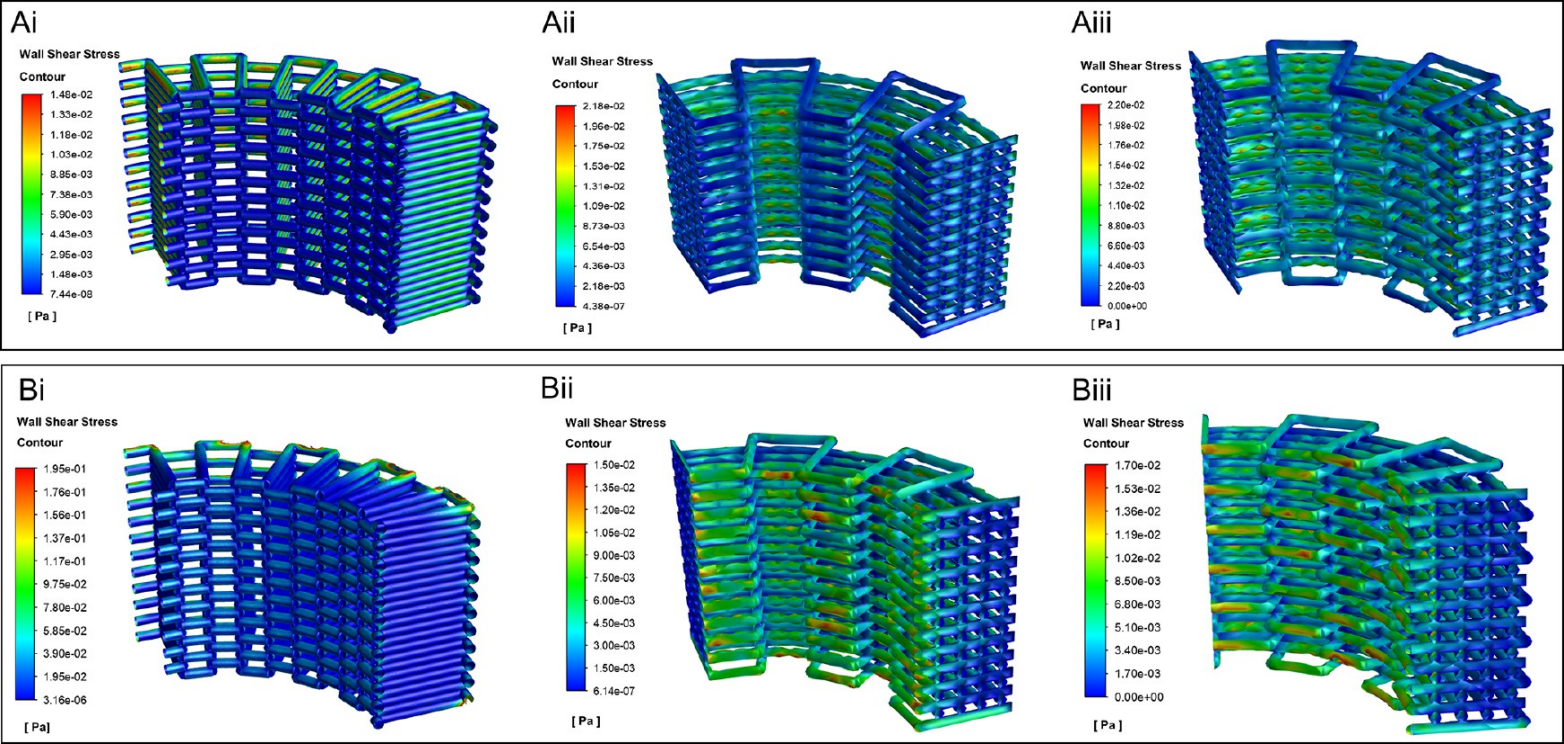
WSS distribution in (A) axial and (B) radial contours of the 3D printed scaffolds of (i) zigzag, (ii) zigzag/spiral, and (iii) zigzag/spiral-shifted patterns.

Figure 7 shows the WSS distribution on the walls of the scaffolds with different patterns in both axial and radial flows, respectively. In the radial direction, the maximum WSS occurred in the zigzag, while the zigzag/spiral had the lowest WSS. In the axial direction, the maximum WSS in the zigzag/spiral and zigzag/spiral-shifted are almost the same and the maximum WSS in the zigzag is the lowest.

The average of WSS and the permeability values of the scaffolds are given in Table 4. Based on the available data in the literature, an average WSS between 0.1 and 10 mPa would be sufficient to initiate osteogenic differentiation of MSCs.^53^ Moreover, an average WSS smaller than 15 mPa is often recommended to differentiate MSCs into osteoblasts and osteocytes in perfusion bioreactor culture systems.^54,55^ The average WSS in all patterns is less than 15 mPa, which reveals that all of the designs have suitable WSS for bone tissue engineering applications.

Moreover, the calculated permeability values for the PCL structures with the same porosity level are from 3.08 ×  10^–9^ to 15.66 ×  10^–9^ m^2^, in the same order of magnitude as those previously reported in the literature for bone tissue.^56,57^

### 3.4 Hybrid Printing

**Figure 8:**
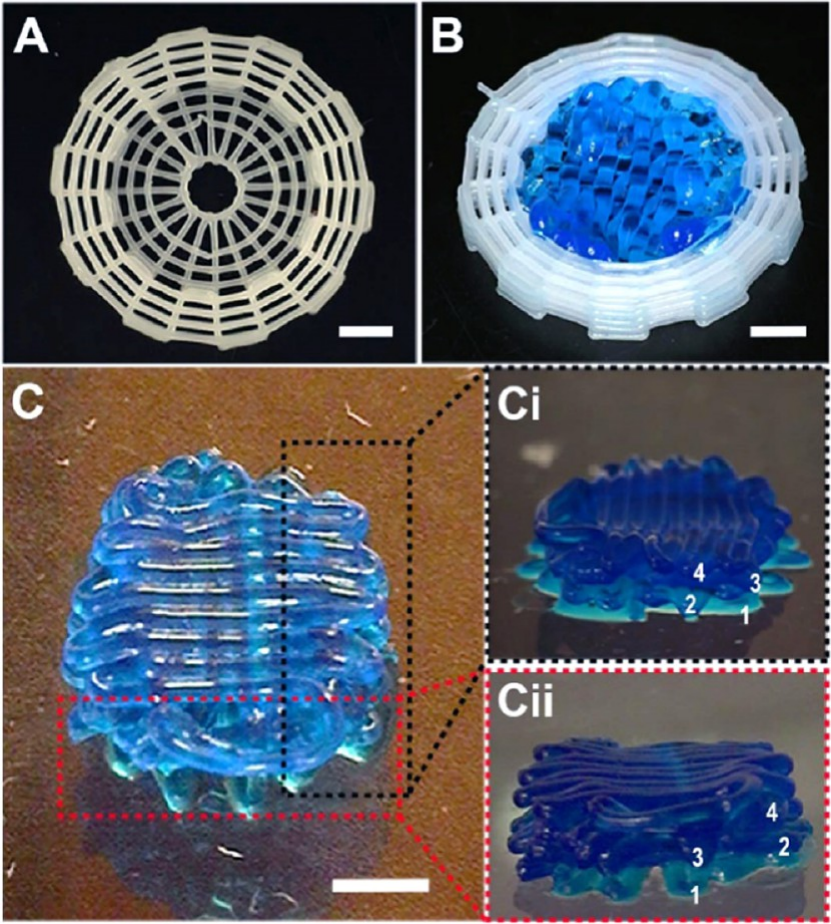
Demonstration of the 3D printed hybrid structures. (A) PCL cage structure, (B) PEG-based bioink bioprinted inside the PCL cage, and (C) multilayer bioprinting of the PEG-based bioink. Four-layers were printed by extrusion of fibers from the microcapillaries. The bioink was separately aspirated in each layer. The side view projections of the multilayered structure are given in (Ci) and (Cii). Scale bars: 2 mm.

According to the mechanical characterization and permeability analysis, the zigzag pattern had the highest compressive stiffness and axial permeability values. However, it showed the lowest radial permeability, which could impede the diffusion of the media from the side of the cage through the cell-laden hydrogel inside. In this context, we selected the zigzag/spiral as the optimum pattern regarding both mechanical properties and permeability. The hybrid 3D bioprinted structure was printed with eight layers of the PCL cage in which four layers of the PEG-based bioink were printed sequentially. A video of a bioprinting hybrid structure at 2× speed is shown as Video S1. To mimic the construction of a segmental large bone defect, a PCL cage structure with 13 mm in diameter and 3 mm thickness was printed in the zigzag/spiral model. The whole structure was divided into 20 points for the zigzag and 5 concentric circles for spiral printing patterns. The first two layers of the PCL cage were printed as a whole circle to ensure that the cage structure would hold the bioprinted hydrogel (Figure 8A). The perimeter of the inner circle of the PCL cage was divided into 60 points and by connecting these points in a meshlike pattern with no gap between the filaments, the printing pattern for the bioink was created. In this way, the bioink was bioprinted completely in contact with the PCL cage. It would assist the shape fidelity of the hydrogel (Figure 8B). The hydrogel filaments were considered to have the same diameter as the capillary inner diameter. To distinguish the consecutive layers, food color at different concentrations was added to the bioinks of each layer (Figure 8C). The printability of the PEG-based bioink was demonstrated in our previous study.^39^ The hydrogel printing path was created for four layers of grid structures with 90° shifts for each layer (Figure 8Ci,ii).

### 3.5 Biocompatibility Evaluation and Osteogenesis

**Figure 9:**
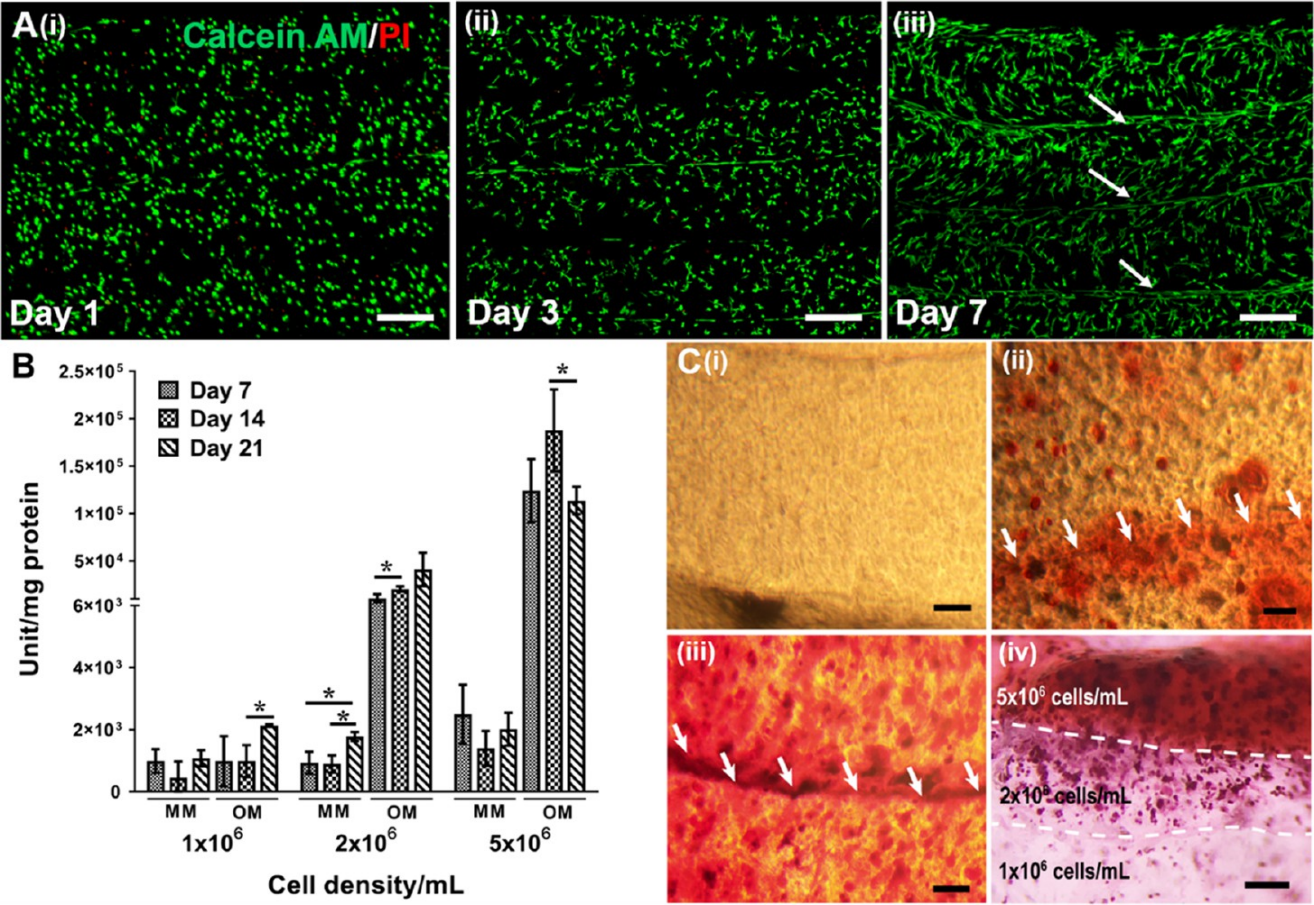
Biocompatibility evaluation of the PEG-based bioink for hBMSCs. (A) Calcein AM (green) and PI (red) staining were performed on (i) day 1, (ii) day 3, and (ii) day 7 after bioprinting to show live and dead cells inside the PEG-based hydrogel. (B) Osteogenic differentiation of hBMSCs in the PEG-based bioink. ALP activity assay was performed to show osteogenic differentiation depending on the varying concentrations of hBMSCs in the bioprinted constructs. Monolayers of PEG-based hydrogel constructs with 1 × 10^6^, 2 × 10^6^, and 5 × 10^6^ hBMSCs/mL were treated with MM and OM for 21 days. The constructs treated with OM were evaluated for osteogenic differentiation while MM-treated cells were used as the control. Student’s *t*-test was used to show the significant levels between the groups (*p < 0.05). The analysis was performed for at least three different samples in a group. (C) Alizarin red staining was performed to show mineralization in monolayers of the PEG-based hydrogel containing 1 × 10^6^, 2 × 10^6^, and 5 × 10^6^ cells (Ci, Cii, Ciii), separately and in a three-layered construct (Civ). Scale bars for (A) and (Civ): 500 μm and (Ci, Cii, Ciii): 200 μm.

The biocompatibility of the PEG-based bioink and the bioprinting process were assessed on NIH 3T3 fibroblast cells in our previous study.^39^ In this paper, we used hBMSCs to induce osteogenesis in the hybrid structure. First, we evaluated the biocompatibility of the bioink for the hBMSCs by monitoring the live and dead cells using Calcein AM/PI staining on days 1, 3, and 7 after bioprinting (Figure 9A). The cells within the bioprinted filaments were seen viable and homogeneously distributed on day 1 (Figure 9Ai). The cells generally have a round-shaped morphology, and their viability is around 90%. The cells maintained their viability on day 3 (Figure 9Aii) and showed a spindle-shaped morphology. On day 7 (Figure 9Aiii), the viable cells were observed with an increased number of the spindle-shaped morphology. Interestingly, the cells with the spindle morphology were aligned toward the regions where the filaments aligned parallel to each other (indicated by white arrows in Figure 9Aiii). This could be because of the micron-sized pores formed between the adjacent PEG-based hydrogel filaments, which should help better nutrient and oxygen diffusion.

The controlled mineralization in the living constructs can increase the potential of the patient-specific functionality of the hybrid structures and improve healing of the critical-size bone defects. The mineralization degree of structures was controlled by varying the cell number in the deposited PEG-based hydrogel filaments in 3D structures in a desired spatial location.^58^ It is well known that three distinct phases are seen during the differentiation of hBMSCs into bone: (1) proliferation of cells in the first 4–5 days, (2) an increase in ALP enzyme activity between 7 and 14 days, and (3) the secretion of collagen type 1 to the extracellular matrix between 21 and 28 days. In the end, a decrease in the ALP enzyme level and accumulation of calcium and phosphate minerals on the collagen matrix secreted into the extracellular matrix are observed.^59^ Considering these stages, ALP activity was first evaluated to understand the effect of the different numbers of cells for the PEG-based hydrogel on the differentiation into bone (Figure 9B). The bioprinted monolayer constructs with varying cell densities 1 × 10^6^, 2 × 10^6^, and 5 × 10^6^ cells/mL were treated with osteogenic medium (OM), and the ALP activity was monitored for 21 days. The samples treated with mesenchymal stem cell basal medium (MM) were used as controls for the comparison. There was no significant difference in the ALP activity of the cells encapsulated in the hydrogels with a 1 × 10^6^ cells/mL initial cell density upon increased incubation time in the control groups. When the samples were treated with OM, a statistically significant increase was observed on day 21 compared to both control groups and the day 7 and 14 ALP activity results. In the samples prepared with a 2 × 10^6^ cells/mL cell density, the ALP activity in the control groups increased significantly on day 21, compared to days 7 and 14. Regardless of incubation times, the OM-treated samples prepared exhibited a significant level of increase in the ALP activity. The ALP activities from day 7 to 14 in the OM-treated samples showed a statistical increase, while no significant change was observed for the later incubation time. Considering the differentiation stages of hBMSCs, we can conclude that the cells in the PEG-based hydrogel prepared with a 2 × 10^6^ cells/mL cell density both proliferate and differentiate into bone between days 14 and 21. In the case of the hydrogels prepared with 5 × 10^6^ cells/mL, the ALP activity of the control samples was obtained possibly due to the increased cell density, yet still in a low value compared to the OM-treated samples. These results suggest that the PEG-based hydrogel does not induce spontaneous hBMSC differentiation into bone. The OM-treated hydrogels prepared with 5 × 10^6^ cells/mL, on the other hand, expressed a high level of ALP activity. The ALP activity reached the maximum level between days 7 and 14 incubation, while a decrease was observed on day 21. According to the differentiation mechanism of hBMSCs, the cells in the PEG-based hydrogel were anticipated in the last stage of the differentiation process, in which the mineralization increased.

The mineralization in the monolayer (i, ii, and ii) and multilayer constructs (iv) after 21 days of incubation in OM is demonstrated in Figure 9C. The alizarin red staining revealed that mineralization strongly depended on the initial cell density encapsulated in the PEG-based hydrogel. The increased density of cells increased the mineralization, which enabled the formation of gradient mineralization in the multilayer constructs. These results are also supported by ALP activity results.

## 4 Conclusions

This study demonstrates the construction of personalized hybrid and gradient structures with unique designs and compositions using a combination of melt extrusion and microcapillary-based 3D printing approaches. The hybrid biomimetic structure was successfully constructed by printing a porous and mechanically strong biodegradable PCL cage on the outside and bioprinting of the molecularly engineered PEG-based bioink with a customized cell density inside the cage. Various architectures of the PCL cage were investigated for optimal mechanical properties and permeability as two critical factors for hybrid scaffolds of critical size bone defects.

According to the compressive stiffness and energy absorbance, the highest values corresponded to the zigzag structure followed by the zigzag/spiral pattern. Moreover, the permeability of the 3D printed PCL cages in both radial and axial directions was also measured as an important factor that indicates how the nutrient/gas can pass through the porous structure.

CFD analysis showed that the zigzag/spiral model had higher permeability compared to the zigzag/spiral-shifted in both directions. Although the zigzag structure showed the highest axial permeability, its radial permeability value was extremely low which could cause problems for the cells deposited inside the cage and even hinder the proliferation and differentiation of the cells in the long term. Hence, we selected the zigzag/spiral model as the most suitable structure for the PCL cage pattern due to its high mechanical strength and permeability both in the radial and axial directions. It can be further studied how these would affect the differentiation of the MSCs loaded into the hydrogel in a long duration of culture.

The molecularly engineered and bioprinted PEG-based hydrogel provided a biologically friendly 3D environment, and hBMSCs maintained their viability and preserved their osteogenic lineage. In addition, the control over the density of cells encapsulated in the hydrogel fibers through the microcapillary-based bioprinting approach demonstrated the capacity of the design for gradient mineralization in microscale precision. It can be utilized for desired and customized differentiation based on different locations of the bone parts. In conclusion, this approach promises the construction of personalized functional implantable structures for critical-size bone defects.

## Supporting Information Available

Bioprinting hybrid structure at 2× speed (Video S1) (MP4)
